# The role of sleep disturbances in associations between early life adversity and subsequent brain and language development during childhood

**DOI:** 10.3389/frsle.2024.1405398

**Published:** 2024-12-04

**Authors:** Hatty Lara, Melissa Nevarez-Brewster, Cori Manning, Matthew J. Reid, Stephanie H. Parade, Gina M. Mason, Darlynn M. Rojo-Wissar

**Affiliations:** ^1^Department of Psychology, University of Arizona, Tucson, AZ, United States; ^2^Department of Psychology, University of Denver, Denver, CO, United States; ^3^Department of Disability and Psychoeducational Studies, University of Arizona, Tucson, AZ, United States; ^4^Department of Psychiatry and Behavioral Sciences, Johns Hopkins School of Medicine, Baltimore, MD, United States; ^5^Department of Psychiatry and Human Behavior, Warren Alpert Medical School of Brown University, Providence, RI, United States; ^6^Bradley/Hasbro Children's Research Center, E.P. Bradley Hospital, Providence, RI, United States; ^7^Sleep for Science Research Lab, E.P. Bradley Hospital, Providence, RI, United States

**Keywords:** early life adversity (ELA), sleep disturbances (SD), childhood, brain development, language development

## Abstract

Sleep disturbances are posited to play a key role in the development of poor mental and physical health outcomes related to early life adversity (ELA), in part through effects on brain development. Language development is critically important for health and developmental outcomes across the lifespan, including academic achievement and emotion regulation. Yet, very little research has focused on the dynamic contributions of ELA, sleep, and brain development on language outcomes. In this mini review, we summarize the current pediatric literature independently connecting ELA and sleep to language development, as well as the effects of ELA and sleep on language-relevant aspects of brain structure and function. We then propose a framework suggesting that sleep disturbances and subsequent effects on brain structure and function may act as key mechanisms linking ELA and language development. Future research investigating the associations among ELA, sleep, brain, and language development will refine our proposed framework and identify whether sleep should be included as an intervention target to mitigate the effects of early life adversity on language development.

## 1 Introduction

Early life adversity (ELA) is a major risk factor for numerous mental and physical health issues from childhood to adulthood (Oh et al., 2018; Zee and Turek, 2006; Duffy et al., 2018). Experiences of ELA include “subjectively perceived threats to the safety or security of the child's bodily integrity, family, or social structures” (Suglia et al., 2018) prior to the age of 18, and can also involve the absence of expected stimuli in cases of deprivation and neglect (Wade et al., 2022). Sleep disturbances are theorized to be a central mechanism linking ELA to poor health outcomes, in part due to their profound effects on multiple neurobiological systems that are also affected by ELA (Fuligni et al., 2021). While a variety of health outcomes have been integrated in these theoretical frameworks, including mood disorders, cardiovascular disease, and diabetes, language development has remained largely overlooked. Here, we summarize evidence in support of language development as another important, but underexamined consequence of ELA that may be partially mediated by sleep disturbances. We first review the literature on language development in the context of ELA and sleep, followed by shared effects of ELA and sleep disturbances on language-related brain structures and functions. We then discuss the plausibility of sleep as a mechanism linking ELA to language-related outcomes during childhood and suggest areas for future research. Much of the research on language development focuses on early childhood, as many fundamental aspects of language development occur within the first 6 years of age (Visser-Bochane et al., 2020; Fenson et al., 1994). Thus, we primarily focus on early childhood, but also broadly cover middle childhood to adolescence.

## 2 Language development in the context of ELA and sleep

Language learning is a prominent aspect of development made up of various constructs (Table 1). It is critical for navigating our social environments and forming social bonds (Carouso-Peck et al., 2021; Fitch, 2005; Locke, 2001), scaffolding our own thoughts and shared concepts (Carruthers, 2002; Lupyan et al., 2007), and for later academic achievement and emotion regulation (Eisenberg et al., 2005). While some language-related abilities such as vowel and voice recognition emerge *in-utero* (Decasper and Fifer, 1980; Moon et al., 2013), language learning is a continuous process, with infants successively identifying and consolidating new words and grammar through exposures in their ambient social environment (e.g., caregivers speaking and pointing) (Visser-Bochane et al., 2020). At school age, language learning priorities shift to reading, grammatical rules (e.g., sentence structure), and writing, which are linked to later occupational and social outcomes (Gaab and Petscher, 2022; Graham, 2022). Although there is a lack of empirical research examining ELA, sleep, and language development together, both ELA and sleep have been independently linked to language development.

**Table 1:** Description of language development and its various constructs.

**Language outcome**	**Definition**
Grammar (Visser-Bochane et al., 2020; Sylvestre et al., 2016; Lum et al., 2015; Coster et al., 1989)	Structural rules of clauses, phrases, and words
Auditory comprehension (Lum et al., 2015)	Ability to understand what is heard
Verbal abilities (Coster et al., 1989; Eigsti and Cicchetti, 2004; Pickett, 2020; Fox et al., 1988)	Ability to vocally use language
Receptive vocabulary (Lum et al., 2017; Di Sante et al., 2019; Qu et al., 2024; Gliga et al., 2023; Knowland et al., 2022; Hernandez-Reif and Gungordu, 2022; Matsuzawa et al., 2001)	Understood words
Expressive vocabulary (Lum et al., 2017; Di Sante et al., 2019; Conway et al., 2021; Pfefferbaum et al., 1994)	Said words
Vocabulary skills (Coster et al., 1989; Eigsti and Cicchetti, 2004)	Ability to understand words, use words, understand word relationships, and use words within context
Verbal intelligence quotients (Kwan et al., 2012)	Measure of acquired knowledge, verbal reasoning, and attention to verbal materials
Literacy skills (Jimenez et al., 2016)	Ability to read, write, speak and listen in order to communicate effectively
Pragmatic skills (Hyter, 2021; Di Sante et al., 2019; Conway et al., 2021)	Language within social contexts, such as responding appropriately, maintaining conversation, and speech turn-taking
Social skills (Lum et al., 2017)	Prosocial behavior such as shows kindness to others when they are upset, communication, cooperation, assertion, responsibility, empathy, engagement, and self-control
Social-linguistic skills (Matsuzawa et al., 2001)	Ability to communicate appropriately toward a specific topic, setting, and relationship
Academic performance (Hyter, 2021; Mills et al., 2011; Ferrara et al., 2023; Palazón-Carrión and Sala-Roca, 2020; Romano et al., 2015; McGregor and Alper, 2015; Botting and Baraka, 2018; Kostović and Jovanov-Milošević, 2006; Ouyang et al., 2019; Stiles and Jernigan, 2010; Kwan et al., 2012; Matsuzawa et al., 2001)	Performance in school subjects

References represent the discussed article(s) that measured the specific language outcome.

### 2.1 Associations between ELA and language

ELA has been consistently associated with poorer child language outcomes, with younger children being particularly vulnerable (Sylvestre et al., 2016; Lum et al., 2015; Matte-Landry and Collin-Vézina, 2020). The most studied aspect of ELA relative to language development is child maltreatment, encompassing sexual, physical, and emotional abuse and neglect (Hildyard and Wolfe, 2002). Generally, abuse and neglect tend to demonstrate comparable effects on language outcomes (Sylvestre et al., 2016; Culp et al., 1991; Allen and Oliver, 1982). However, in early childhood, neglect has specifically been related to poorer pragmatic skills (Hyter, 2021). Overall, child maltreatment in early childhood is associated with multiple dimensions of language development, including lower vocabulary skills, poorer grammar (Coster et al., 1989; Eigsti and Cicchetti, 2004), and impaired auditory comprehension and verbal abilities (Lum et al., 2015; Coster et al., 1989; Eigsti and Cicchetti, 2004; Pickett, 2020; Fox et al., 1988). Work extending beyond early childhood has also linked maternal intimate partner violence, in addition to child maltreatment, to poorer receptive, expressive, and social skills (in 5–12-year-olds) (Lum et al., 2017), and pragmatic and reading skills (in 10-year-olds) (Di Sante et al., 2019; Conway et al., 2021). Several reviews support the above, demonstrating links between ELA and communication, reading abilities, and academic performance and achievement in children of all ages (Hyter, 2021; Mills et al., 2011; Ferrara et al., 2023; Palazón-Carrión and Sala-Roca, 2020; Romano et al., 2015; Snow, 2020).

More recent studies have also examined the effects of *cumulative* exposure to adverse childhood experiences (ACEs), including household dysfunction and child maltreatment, on language development. One study found a graded relation between number of ACEs and poorer vocabulary scores (1–3-year-olds) (McKelvey et al., 2017), and another found that those with ≥3 ACEs had below-average language and literacy skills (2–5-year-olds) (Jimenez et al., 2016). In contrast, in a study of children (Mean age = 6.9) with any of 55 traumatic event types, those who developed specific language impairments were 1.46 × more likely to have been exposed to physical trauma specifically, but were not more likely to have experienced a greater number of ELA types (Selin et al., 2022). In summary, both maltreatment and number of ACEs confer risk for compromised language development across different domains (Stewart-Tufescu et al., 2022; Qu et al., 2024), but effects may differ based on characteristics of the ELA (e.g., type, intensity) and language outcome being assessed. Further, while most research on ELA and language has been conducted in early childhood, studies in middle childhood and adolescence suggest that ELA can affect language outcomes beyond early childhood (Mills et al., 2011; Selin et al., 2022).

### 2.2 Associations between sleep and language

Sleep is also implicated in language development (Dionne et al., 2011; Mohammed et al., 2021). In this mini review, we focus on aspects of sleep that have been most robustly linked with ELA (Brown et al., 2022; Schønning et al., 2022) specifically, sleep disturbances related to insomnia (Mindell et al., 1999). Sleep disturbances related to pediatric insomnia can include trouble falling or staying asleep, and related difficulties with sleep consolidation (i.e., consolidating sleep into one continuous overnight interval), short sleep duration, and poor sleep quality (Brown and Malow, 2016; Owens and Mindell, 2011). Children with shorter overnight sleep or less consolidated sleep also tend to have longer daytime naps in early childhood (Jones and Ball, 2014; Ordway et al., 2020). Given that several fundamental language milestones occur when infants and toddlers are still napping (Visser-Bochane et al., 2020), most studies in these age groups have focused on the benefits of naps for language (Simon et al., 2017; Horváth et al., 2016; Hupbach et al., 2009; Gómez et al., 2006; Sweeney et al., 2023; Sandoval et al., 2017; Friedrich et al., 2017); however, the effects of naps on immediate and enduring sleep disturbances are unclear. Studies on sleep consolidation in infancy demonstrate that more consolidated overnight sleep and better parent-led sleep hygiene are associated with better vocabulary, receptive language, and overall communicative development in toddlerhood (Qu et al., 2024; Gliga et al., 2023; Knowland et al., 2022; Hernandez-Reif and Gungordu, 2022). Short sleep duration across the first 3 years of life, and among preschool and school-aged children, has consistently been linked with poorer cognitive scores (Smithson et al., 2018), and worse vocabulary and academic performance overall (Friedrich et al., 2017; Gliga et al., 2023; Knowland et al., 2022; Hernandez-Reif and Gungordu, 2022; Smithson et al., 2018). With regard to sleep disturbances more broadly, studies from infancy to adolescence have shown that multiple types of sleep disturbances (e.g., difficulty falling asleep or staying asleep) are independently associated with poorer language skills and lower verbal intelligence quotients (McGregor and Alper, 2015). For example, disturbances in sleep onset, duration, and nighttime-awakenings predicted decreased expressive, receptive, and social-linguistic skills in 3–18-year-olds (Botting and Baraka, 2018). Together, these studies emphasize that early sleep insufficiency, as well as disrupted sleep, may have an enduring negative impact on language abilities.

Few studies have examined associations between sleep measured with polysomnography (PSG) and language development. One study found that greater neonatal electroencephalogram (EEG) connectivity during non-rapid-eye-movement (NREM) sleep was associated with more advanced vocabulary scores at 18 months (Shellhaas et al., 2022). In another study of 8-year-olds, mean spindle frequency was negatively linked to executive functions, including planning ability and working memory (Chatburn et al., 2013). A study of 10-year-olds with dyslexia showed that greater spindle density during NREM sleep was positively correlated with reading abilities (Bruni et al., 2009). Finally, developmental increases in frontal slow spindle density (11–13 Hz) from ages 8–11 to 14–18 years were positively associated with general cognitive abilities at 14–18 years, including vocabulary skills (Hahn et al., 2019). Additional studies are needed to elucidate how PSG measures of sleep are linked to ELA-related subjective sleep disturbances, and in turn language development.

## 3 Shared mechanisms: effects of ELA and sleep on brain structure and function

Brain development in early human life is characterized by abundant dendritic arborization, synaptogenesis and connectivity, as well as reorganization and overproduction of cortical circuits (Kostović and Jovanov-Milošević, 2006; Ouyang et al., 2019; Stiles and Jernigan, 2010). Synaptic density in white and gray matter within the frontal cortex peaks by the second year of life (Kwan et al., 2012). Increased axonal density and myelination in white matter connections also occur (Matsuzawa et al., 2001). Inevitably, this orchestrated growth culminates in an adult-like brain structure (Pfefferbaum et al., 1994) and promotes environment-directed learning, language acquisition, and emotion processing later in life (Keunen et al., 2017).

### 3.1 Brain mechanisms underlying language

Early language learning is, arguably, a whole-brain endeavor. While some theories—largely based on adult findings—have proposed that specialized neocortical areas are responsible for specific language abilities (Friederici, 2006), more recent perspectives elucidate the importance of more domain-general brain networks in language, including social-motivational limbic brain systems (Syal and Finlay, 2011), hippocampal and temporal memory-related networks (Goldstein et al., 2010), and broader sensory and motor pathways (Mason et al., 2019; Paterson et al., 2006).

In new word learning, typically-developing infants must map auditory input they receive from caregivers with co-occurring visual and/or tactile sensations across multiple experiences (Mason et al., 2019). Since language learning is an experience-dependent process, caregivers facilitate infants' learning by providing exaggerated (“infant-directed”) and/or simplified speech that is reliable and appropriately timed (Elmlinger et al., 2023, 2019; Fernald and Simon, 1984). Even so, infants must be able to attend to and detect predictable patterns in caregivers' feedback (e.g., how frequently/promptly/reliably caregivers respond to infant behaviors) (Elmlinger et al., 2019; Tamis-LeMonda and Bornstein, 1989; Venditti et al., 2023), and find social exchanges rewarding to continue engaging and learning from others (Syal and Finlay, 2011; Venditti et al., 2023). Given this multifaceted pathway to language learning, it is reasonable that multiple brain networks, including those involved in sensory processing, memory, and reward, would be implicated in language.

At later ages, when language becomes more of an academic undertaking in formal educational settings, brain areas and networks subserving attention, memory consolidation, and cognitive flexibility may be most at play for promoting academic success in language-related skills (e.g., writing composition and grammar). Such areas include the dorsolateral prefrontal cortex (DLPFC) and anterior cingulate cortex (ACC) (Skeide and Friederici, 2016; Opitz and Friederici, 2003; Hertrich et al., 2021; Piai et al., 2013). Broca's and Wernicke's areas are also well-known for language comprehension and production (Rosselli et al., 2014).

During adolescence, sustained attention, working memory capacity, and strategic memory processes continue to refine due to changes in the prefrontal cortex (PFC) and hippocampus, enabling adolescents to engage in complex cognitive demands in academic settings (Calabro et al., 2020). Effective memory processes enable students to encode, store, and retrieve information necessary for academic success, including factual knowledge, problem-solving strategies, and conceptual understanding (Swanson and Alloway, 2011). In high schoolers, higher density of gray matter in the left DLPFC is associated with higher academic achievement (Wang et al., 2017). From infancy to adolescence, these brain changes reflect the progressive development of language abilities and cognitive skills essential for effective communication, literacy, and academic achievement.

### 3.2 Associations between ELA and the brain

Across species, ELA is associated with alterations in neurodevelopment (Berman et al., 2022; Callaghan and Tottenham, 2016; Luby et al., 2020; Short and Baram, 2019), which may contribute to subsequent impairments in language learning. In rodents, poverty as an ELA can be simulated by limiting bedding and nesting materials, and inconsistent parenting can be represented by unpredictable behaviors from the rodent mother (i.e., dam). In controlled experiments, these forms of ELA in rodents are linked to alterations in corticolimbic and frontolimbic white matter (e.g., uncinate fasciculus), reductions in hippocampal volume and intra-hemispheric hippocampal white matter, accelerated development of whole-cortex connectivity, and changes to neural circuitry in the amygdala and ventral and dorsal striatal regions (Short and Baram, 2019; Molet et al., 2016; Birnie et al., 2020; Chan et al., 2024). Behaviorally, such changes in brain structure and function are related to anxiety-like behaviors and emotion processing, executive and cognitive functions, memory consolidation and recall, and reward sensitivity (Birnie et al., 2020; Van Petten, 2004).

Randomized controlled trials and observational studies in school-aged children (8–11-years-old) indicate that ELA in the form of institutionalized rearing relates to greater amygdala white matter volume (Tottenham et al., 2010) and accelerated structural development of amygdala-medial PFC connectivity (Gee et al., 2013), both of which are implicated in emotion processing and abnormally heightened threat detection (Tottenham et al., 2010; Ribeiro et al., 2023). On a conceptual level, such alterations in emotion processing could influence language learning by altering the interpretation and processing of non-verbal cues that aid language development. Heightened threat detection could also inhibit language learning by limiting attention toward language-promoting stimuli, and consequently their processing and encoding into memory. Both child maltreatment and unpredictable maternal signals have been linked to greater brain myelination in 9–11-year-old children, and less myelination in 18–22-year-olds, in a major frontolimbic tract (uncinate fasciculus) (Hanson et al., 2015; Granger et al., 2021). Opposing findings regarding the magnitude of myelination in these two studies may be partially due to age and developmental maturity of the brain at assessment (Gee et al., 2013; Ribeiro et al., 2023; Hanson et al., 2015). Nevertheless, both greater and less myelination have been related to compromised emotion processing, suggesting that adequate myelination within sensitive windows of development is required for optimal language function (Banihashemi et al., 2020; Mincic, 2015; Modi et al., 2013). Overall, the link between ELA and brain structure and function is undisputed, and there is now a push to identify intermediary pathways linking them (Shackman and Gee, 2023), including sleep-related variables such as insomnia (Fuligni et al., 2021; Luby et al., 2020).

### 3.3 Associations between sleep and the brain

Recent findings highlight the critical role of pediatric sleep in neurodevelopment, with implications for language learning (Mason et al., 2021; Alrousan et al., 2022; Galván, 2020). Developmental experiments in nonhuman animals show that adequate sleep duration promotes molecular and neural network adaptation (Bridi et al., 2015), while insufficient or disturbed sleep gives way to maladaptive growth and development, such as exacerbating the effects of visual deprivation on the development of visual-cortical systems (Frank et al., 2001). Further, sleep in earlier stages of childhood may be more robustly associated with brain structure and function (Lokhandwala and Spencer, 2022). For example, in a sample of 200 children (4–8-years-old), longer 24-hr sleep duration predicted larger hippocampal subfield volumes, but only in the younger cohort (4–6 vs. 6–8 years) (Riggins and Spencer, 2020). The accumulation of findings has enabled scholars to identify putative neurobiological pathways in the association between early life sleep disturbances and compromised cognitive development (Alrousan et al., 2022; Jan et al., 2010; Mason and Spencer, 2022).

Changes in sleep-related brain activity (e.g., sleep spindles and slow oscillations) relevant for brain structure and dendritic density are some mechanisms through which sleep disturbances in early life (Lokhandwala and Spencer, 2022; Kurth et al., 2010; Pittner et al., 2023) may lead to impaired emotion processing, cognition, and memory (Kurth et al., 2015; Lopez et al., 2010), which may in turn impact language. Illustrating the relations between early sleep disturbance and brain development, one study reported that insufficient sleep in 6–9-month-olds prospectively predicted smaller white matter volume at 1-year (Pittner et al., 2023). This finding suggests that sleep disturbances in infancy may affect myelination (Frank et al., 2001) and communication across brain regions that facilitate complex cognitive processes, such as executive functioning and emotion processing (Lokhandwala and Spencer, 2022). Similarly, in a longitudinal cohort study of 720 six-year-old children, pediatric sleep disturbances, such as trouble falling asleep and resisting going to bed, were associated with decreased gray matter volume (Kocevska et al., 2017), which may be associated with later psychopathology (Wise et al., 2017). On a functional level, a study conducted in 5–9-year-olds found that shorter sleep duration on weekdays was associated with lower amygdala-ACC resting state functional connectivity (Hansen et al., 2024), which is also related to emotional processing (Etkin et al., 2015). These findings extend to school-age children and adolescents, as summarized by a review of this work demonstrating associations between a variety of sleep disturbances (e.g., in quality, duration, and variability) and brain connectivity in areas related to memory, attention, and reward processing (Dutil et al., 2018). Notably, some findings link *changes* in sleep from infancy to early childhood to white and gray matter (Pittner et al., 2023; Kocevska et al., 2017), suggesting that developmental sleep trajectories may be an important and understudied predictor of neurodevelopment that may subsequently affect language.

## 4 Sleep disturbances as a pathway linking ELA and language outcomes

**Figure 1:**
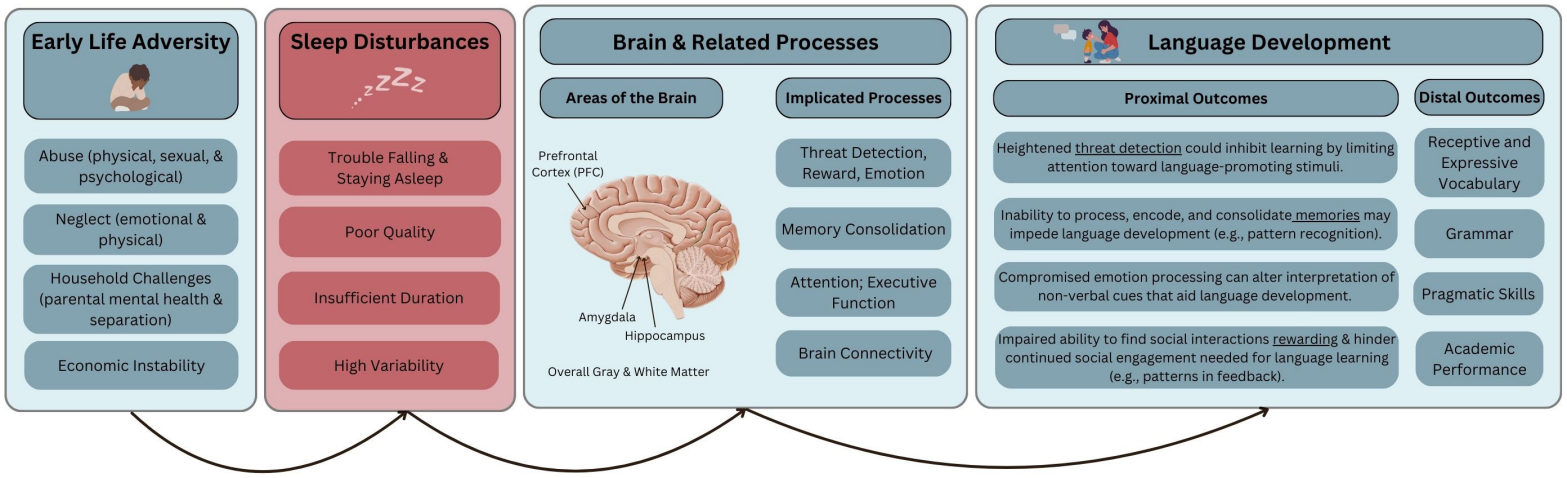
Conceptual model of sleep's potential role as a mechanism linking early life adversity to impaired language development via alterations to brain structure and function. We acknowledge that associations among these constructs are complex and often bidirectional. However, the arrows represent the theoretical model focused on in this review.

Due to overlap in the negative neurobiological and mental and physical health consequences of both ELA and sleep disturbances, a previous theoretical model suggests that sleep disturbances are a key mechanism linking ELA to poor health outcomes across the lifespan (Fuligni et al., 2021). We extend this theoretical model to include language development as an outcome, with a focus on childhood (see Figure 1 for a schematic representation of our framework). Thus far, we have summarized the effects of ELA and sleep disturbances on both brain structure and function, and language-related outcomes. Additional support of this model comes from the accumulating literature on associations between ELA and sleep disturbances.

### 4.1 Associations between ELA and sleep

ELA has been linked with several behavioral sleep disturbances during childhood and adolescence (Brown et al., 2022; Schønning et al., 2022). The most robust associations have been with insomnia and insomnia-related symptoms, including difficulty falling and staying asleep (Brown et al., 2022; Schønning et al., 2022). A recent meta-analysis showed that child maltreatment is associated with four times greater odds of insomnia symptoms among children 5–18 years of age (Schønning et al., 2022). In a nationally representative study of children born to unwed parents, number of caregiver-reported ACEs through age 9 was associated with trouble falling asleep ≥3 times per week at age 15 (Rojo-Wissar et al., 2021). A similar epidemiologic study showed that adolescents with ≥1 ACE were 1.93 times more likely to develop an insomnia disorder compared to those with no ACEs, and those with ≥5 ACEs were at 3 times greater risk (Wang et al., 2016). Evidence has also demonstrated links of ELA with shorter sleep duration, lower sleep efficiency, and nightmares in children (Brown et al., 2022; Schønning et al., 2022; Sadikova and Mazurek, 2024). In particular, 6–36-month-olds from socioeconomically disadvantaged homes have shown shorter than recommended sleep duration for their age (Ordway et al., 2020; Mindell and Williamson, 2018).

Very few studies have examined associations between ELA (in the form of abuse or institutionalized rearing) and sleep measured via actigraphy in children. Most of these studies show associations with worse sleep efficiency but are somewhat mixed with regard to other sleep parameters (Glod et al., 1997; Tininenko et al., 2010; Sadeh et al., 1995). There are no known studies examining the relationship between ELA and sleep measured by PSG in children. However, there are a few studies in adults that suggest ELA, such as perceived lack of safety or exposure to traumatic events (e.g., abuse), may be associated with greater fragmentation in rapid-eye-movement (REM) sleep and less slow spindle density (Insana et al., 2012; Nielsen et al., 2019).

### 4.2 Sleep disturbances as a key pathway

Strong evidence links ELA with the development of sleep disturbances, and sleep disturbances with language-relevant alterations in brain structure and function; nonetheless, limited research focuses on ELA, brain structure and function, sleep disturbances, and language outcomes altogether. However, a few studies provide initial evidence for the potential role of sleep as a mechanism linking ELA to impaired language development via alterations to brain structure and function. One study among young adults (18–19-years) found that sleep efficiency partially mediated the association between retrospectively reported child maltreatment and reductions in gray matter hippocampal volume (Teicher et al., 2017), which has been linked to later language outcomes (Deniz Can et al., 2013; Bellander et al., 2016). Similarly, another study found that sleep quality in middle and high schoolers partially mediated the relationship between ELA and academic achievement (e.g., English proficiency) (Qu et al., 2024). There is also evidence that maternal sleep disturbances may mediate or moderate associations between maternal ELA and offspring emotion processing (Ciciolla et al., 2022) and epigenetic aging (Sosnowski et al., 2024), supporting theoretical work positing that sleep during sensitive developmental periods (e.g., infancy and childhood) may act as a conduit mitigating or exacerbating the effects of ELA on health and language outcomes (Fuligni et al., 2021). Given robust theoretical evidence but scant empirical evidence focusing on the dynamic relations among ELA, sleep disturbances, brain structure and function, and language, it is critical for future studies to examine these developmental factors simultaneously.

## 5 Limitations of current literature and future directions

The present literature has allowed scholars to understand the effects of ELA on brain structure and function, sleep disturbances, and language development separately; however, there are limitations in the current body of knowledge. First, though some work has begun to ascertain how ELA types may differentially affect neurodevelopmental outcomes (McLaughlin et al., 2014; Gee, 2021), relatively little research has considered important characteristics of ELA above and beyond exposure, including ELA timing, type(s), chronicity, severity, and cumulative burden (but see Selin et al., 2022; Bethell et al., 2017; Berman et al., 2022). Future studies are needed to determine the relative importance of these ELA characteristics, and how they interact to affect sleep and language development. Second, although some interventions such as home visiting programs have been shown to prevent child maltreatment (Han and Oh, 2022), promote healthy sleep (Schwichtenberg et al., 2019; Kuhn and Elliott, 2003; Fangupo et al., 2021), and improve language development (Peacock et al., 2013; Henwood et al., 2020; Pentimonti et al., 2022) they have not been examined all together in the same study. Additionally, studies focusing on specific clinical populations, including children with neurodevelopmental delays such as autism spectrum disorder, are needed to further elucidate the full effects of ELA on language outcomes (Sadikova and Mazurek, 2024). Future studies examining the effects of pediatric sleep interventions following ELA on language development in both general and clinical populations are warranted.

In addition to brain development, the Hypothalamic-Pituitary-Adrenal (HPA) axis and immune systems are pathways that have been implicated as mechanisms linking ELA and sleep disturbances to poorer mental and physical health outcomes (Fuligni et al., 2021). Here we focused on reviewing the sleep and brain-related pathways which are likely the most relevant to language development specifically, but future research should also explore the potential roles of the HPA-axis and immune system. Finally, previous evidence demonstrates that racial/ethnic disparities exist in ELA exposure (Suglia et al., 2020; O'Connor et al., 2020) and poor sleep outcomes (El-Sheikh et al., 2022; Billings et al., 2021; Jean-Louis and Grandner, 2016), and the accumulation of disparities across these systems may result in disparities in language development (Zuckerman et al., 2014; Justice et al., 2020). Future studies seeking to understand the multilevel disparities in place that affect ELA, sleep disturbances, brain structure and function, and language outcomes are crucial for effectively promoting language development in children from all families and backgrounds. Overall, longitudinal studies assessing ELA, sleep disturbances, and their combined effects on brain and language development are needed to further develop and elucidate the theoretical framework that sleep disturbances are an important pathway to consider in the associations between ELA and brain and language development.

## 6 Conclusion

The intricate connections among ELA, sleep disturbances, and brain structure and function underscore the multifaceted nature of overall development and its long-term implications for health and language outcomes specifically. Language development, from infancy through adolescence, engages an interplay of brain regions and networks, encompassing sensory processing, memory, reward, attention, and executive function. ELA, historically characterized as child maltreatment and ACEs, is consistently linked to compromised language skills across various developmental stages (Sylvestre et al., 2016; Lum et al., 2015), affecting vocabulary, grammar, pragmatic skills, and academic achievement. Likewise, sleep disturbances, spanning from infancy to adolescence, have shown enduring effects on language abilities, with insufficient or disrupted sleep predicting lower cognitive scores (Cheng et al., 2021), and worse performance on vocabulary measures (McGregor and Alper, 2015; St. Laurent et al., 2023), and academically overall (Ravid et al., 2009; Williamson et al., 2020). Our proposed framework considers both subjective and objective measures of sleep. However, given the lack of research using actigraphy and polysomnography, future studies should incorporate objective sleep assessments to evaluate the impact of measurement methodology and advance this framework.

Understanding the intricate and dynamic associations among ELA, sleep disturbances, and brain structure and function is imperative for promoting healthy language development in children of all backgrounds. Brain structures that play critical roles in emotion processing, memory, and cognitive functions are notably affected by both ELA (Luby et al., 2020; Gee et al., 2013; Dutil et al., 2018) and sleep disturbances (Mason and Spencer, 2022; Pittner et al., 2023; Kurth et al., 2015), suggesting converging neurobiological pathways that may mediate subsequent effects on language development. However, there is a scarcity of studies examining all of these together, and those that do, haven't considered language as an outcome. Moving forward, future research addressing these limitations by exploring mechanistic models and elucidating the role of sleep interventions to address language deficits associated with ELA, is needed. Additionally, considering disparities in access to healthcare and resources, particularly among underserved populations, is paramount for developing targeted interventions that mitigate the adverse effects of ELA and sleep disturbances on language development.
